# Influence of Anthocyanin Expression on the Performance of Photosynthesis in Sweet Orange, *Citrus sinensis* (L.) Osbeck

**DOI:** 10.3390/plants12233965

**Published:** 2023-11-24

**Authors:** Alissar Cheaib, Lamiaa M. Mahmoud, Christopher Vincent, Nabil Killiny, Manjul Dutt

**Affiliations:** 1 Department of Plant Pathology, Citrus Research and Education Center, Institute of Food and Agricultural Sciences, University of Florida, Lake Alfred, FL 33850, USA; acheaib@ttu.edu; 2 Department of Horticultural Sciences, Citrus Research and Education Center, Institute of Food and Agricultural Sciences, University of Florida, Lake Alfred, FL 33850, USA; lamiaa.mahmoud@ufl.edu (L.M.M.); civince@ufl.edu (C.V.); manjul@ufl.edu (M.D.)

**Keywords:** anthocyanins, *VvmbA1*, photosynthesis, gas exchange, chlorophyll fluorescence, carotenoids, Rubisco

## Abstract

Anthocyanins are a class of natural pigments that accumulate transiently or permanently in plant tissues, often in response to abiotic and biotic stresses. They play a photoprotective role by attenuating the irradiance incident on the photochemical apparatus and quenching oxyradicals through their powerful anti-oxidative function. The objective of the current study is to understand the impact of introducing *Vitis vinifera* mybA1 (*VvmybA1*) in ‘Hamlin’ sweet orange trees on various aspects, including photosynthetic performance, pigment composition, and gene expression related to photosynthesis and light harvesting. We describe the relationship between anthocyanin accumulation and photosynthetic measurements in genetically modified ‘Hamlin’ sweet orange trees expressing the grapevine-derived Vitis vinifera mybA1 (*VvmybA1*). The juvenile leaves of transgenic plants displayed an intense purple color compared to the mature leaves, and microscopic visualization showed anthocyanin accumulation primarily in the leaf epidermal cells. Under optimal growth conditions, there were no significant differences in leaf gas exchange variables, suggesting normal photosynthetic performance. The chlorophyll fluorescence maximum quantum yield of PSII was slightly reduced in *VvmybA1* transgenic leaves compared to the performance of the control leaves, while the total performance index per absorbance remained unaffected. Comparison of the chlorophyll and carotenoid pigment contents revealed that chlorophyllide *a* and carotenoid pigments, including trans-neoxanthin, trans-violaxanthin, cis-violaxanthin, zeaxanthin, antheraxanthin, and total xanthophylls were enhanced in *VvmybA1* transgenic leaves. Although there were no significant changes in the rates of the gas exchange parameters, we recorded a high relative expression of the ribulose-1,5-bisphosphate carboxylase/oxygenase large subunit (RuBP) and rubisco activase (RCA) in the mature leaves of transgenic plants, indicating activation of Rubisco. Our findings confirm an efficient photoacclimation of the photosynthetic apparatus, allowing the transgenic line to maintain a photosynthetic performance similar to that of the wild type.

## 1. Introduction

Anthocyanins are glucosides of the anthocyanidins derived from a specific flavonoid pathway, responsible for the red-to-blue pigmentation of leaves, flowers, and fruits [1]. In nature, anthocyanins are permanently synthesized in some species, while in others, they may only appear in juvenile leaves as a transient trait that gradually disappears in photosynthetically mature tissues. Additionally, multiple biotic or abiotic stress factors significantly induce anthocyanin biosynthesis in plant tissues. These stress factors include wounding, UV-B radiation, high light exposure, temperature stress, transplant shock, nutrient deficiency, insect herbivory, and pathogen attack [2,3]. The eco-physiological functions of anthocyanins in response to unfavorable conditions are exclusively ascribed to (i) photoprotection from UV and visible light, (ii) anti-oxidative function, and scavenging the overproduction of reactive oxygen species (ROS) induced during stress, (iii) protection against herbivores and pathogens [4], and (iv) carbohydrate sinks that regulate photosynthesis and prevent starch accumulation under stress conditions [5]. These functions are part of an integrated defense network that allows a successful counteraction to lead to detrimental effects following severe exposure to light or oxidative stress [6].

The anthocyanin biosynthesis pathway has been intensively studied to elucidate many essential applied biological and genetic processes [7]. They are synthesized in the cytosol and are derived from the phenylpropanoid pathway [8]. Several enzymes work sequentially to enhance anthocyanin biosynthesis, starting with phenylalanine ammonia lyase (PAL), cinnamate 4-hydroxylase (C4H), chalcone synthase (CHS), and flavanone 3-hydroxylase (F3H) in the phenylpropanoid pathway. Dihydroflavonol 4-reductase (DFR), anthocyanidin synthase (ANS), and UDP-glucose, flavonoid 3-O-glucosyltransferase (UFGT), induce reactions leading to flavonoids and anthocyanin biosynthesis [9]. The final steps involve the action of enzymes like anthocyanidin synthase and anthocyanidin reductase, resulting in the formation of anthocyanidins—the colorful pigments responsible for the red, purple, and blue hues observed in various plant tissues. The regulation of these structural genes is well-studied and involves three main families and several transcription factors. R2R3-MYB and basic helix-loop-helix (bHLH) domains and conserved WD40 repeats (WDR) control anthocyanin biosynthesis in plant tissues [10].

R2R3-MYBs primarily regulate phenylpropanoid metabolism in several plants [11]. Genetic engineering via anthocyanin regulatory MYBs leads to the alteration of anthocyanin pathway enzymes and the regulated proteins [12,13]. The *IbMYB1* gene from sweet potato, the *MdMYB10* gene from apple, and the *PAP1D* gene from Arabidopsis induce anthocyanin accumulation [14,15,16]. Genetically modified anthocyanin-enriched lines of ‘Mexican’ lime (*Citrus aurantifolia*) were developed by constitutively overexpressing the blood orange-derived Ruby gene from Moro or *VvmybA1* from grape vine [12,13]. Anthocyanin-producing ‘Mexican’ lime plants produced fruits with dark red pulp rich in antioxidants, lower in titratable acidity, and slightly lower in sugars [17]. *VvmybA1* expression altered the metabolic pathways and contributed to the additive nutritious value and quality characteristics of the fruit juice [17].

Photosynthesis is highly responsive to irradiance. The efficiency of photosynthesis is limited by various theoretical factors, such as the efficiency of absorption of light energy and the transduction efficiency into biomass. Photosynthesis is affected by many factors, including light intensity, Rubisco activity, and stomatal conductance. In full sun conditions, the light-harvesting pigment–protein complexes can capture higher energy than can be captured by photochemistry or photosynthesis [18].

Broad studies have been conducted to investigate the photosynthetic performance of natural anthocyanins in leaves or the induced expression in genetically modified plants. Anthocyanin-rich leaves exhibit shade properties and are less prone to dynamic photoinhibition in many crops [3]. The major protective role of anthocyanin depends on photosystem protection from light damage by absorbing more photons that would otherwise excite chlorophyll pigments [19], thus protecting chloroplasts from photoinhibition by diminishing energy flux through chlorophylls [20,21].

Citrus is one of the genera that expresses anthocyanin biosynthesis traits in some species. It naturally occurs in blood oranges (*Citrus sinensis* (L.) Osbeck) by activating a retrotransposon inserted in the promoter of the Ruby gene induced following cold conditions [22]. The scarcity of the necessary environmental stimulus to activate the retroelement causes the absence of this trait in warm tropical and sub-tropical climates [23,24]. It also presents in various plant tissues of citron (*Citrus medica* L.), lemon (*Citrus limon* (L.) Burm. f.), finger lime (*Citrus australasica),* and other citrus species [25,26]. Few studies have focused on the effect of anthocyanin biosynthesis on the overall efficiency of the photosynthetic machinery in citrus plants.

The primary objective of the current study was to investigate the ability of the photosynthetic apparatus to adapt and acclimate to reduced light absorption resulting from the accumulation of anthocyanins under ideal growth conditions. To achieve this, we measured the levels of gas exchange, various chlorophyll fluorescence parameters, and the content of pigments such as chlorophylls, carotenoids, and xanthophylls in leaves enriched with anthocyanin, comparing the results with those from the wild-type control plants. Additionally, we examined the relative expression of genes related to ribulose-1,5-bisphosphate (RuBP) carboxylase/oxygenase and Rubisco activase (RCA) in the transgenic plants in comparison to the control plants, all under the optimal temperature.

## 2. Materials and Methods

### 2.1. Plant Transformation and Vector Construction

Transgenic lines of the ‘Hamlin’ (*Citrus sinensis*) variety expressing the *VvmybA1* (AB097923) transgene were generated under the control of a 35S promoter using Agrobacterium-mediated transformation, as described earlier [12]. The transformed stems were placed in regeneration media, and the selected transgenic plants were micro-grafted onto Carrizo citrange rootstock [27]. A transgenic line with enhanced visual anthocyanin production was selected. The greenhouse-acclimated tree was clonally propagated by budding onto Swingle citrumelo rootstock. The plants were grown in a PRO-MIX soil-less medium (Premier Tech Horticulture, Quakertown, PA, USA) in standard square “citripots” and fertilized by biannual Harrell’s^®^ 16–5–10 nursery controlled-release fertilizer (CRF) mix. The plants were irrigated biweekly and maintained at relative humidity (RH) 65 ± 5% and 30 ± 2 °C with a 16 h light/8 h dark photoperiod. All subsequent data were collected from two-year-old trees maintained in climate-controlled growth chambers located at the Citrus Research and Education Center. All leaf samples were collected from the 4th to 8th fully expanded leaves below the apical meristem for all the studied parameters.

### 2.2. Molecular Analysis of Transformants through Quantitative Real-Time Reverse Transcriptase-PCR (qRT-PCR)

We analyzed the expression of *VvmybA1*, *RuBP*, and *RCA* transcripts in the transgenic line and the wild-type control using real-time PCR. Leaf samples were collected for total RNA extraction using the TRIzol reagent (Ambion, Life Technologies, Carlsbad, CA, USA) and Direct-zol™ RNA Miniprep (Zymo Research, Irvine, CA, USA) according to the manufacturer’s protocol. The cDNA was synthesized at 42 °C for 60 min using the RevertAid First Strand cDNA Synthesis Kit (Thermo Fisher Scientific, Waltham, MA, USA). Forty ng of cDNA were used for quantitative real-time reverse transcription (qRT-PCR) performed in the StepOnePlus Real-Time PCR system (Applied Biosystems, Thermo Fisher Scientific, Waltham, MA, USA) with the Pow SYBR^®^ Green PCR Master Mix (Applied Biosystems, Foster City, CA, USA) at the following conditions: 95 °C for 10 min, 40 cycles at 95 °C for 15 s, and 60 °C for 60 s. Each sample was assessed in triplicate to ensure accuracy. The expression level of each target gene was calculated by the 2^−∆∆CT^ method of Livak and Schmittgen [28]. Gene expression analysis was performed for three plants from each phenotype and three technical replicates for each plant (*n* = 3). Data were analyzed using the Applied Biosystems software version 2.0.6. β-actin was used for the normalization of gene expression. The primer sequences are listed in Table 1.

### 2.3. Microscopic Imaging

To examine the localization of the pigments in the plant tissues, leaves were collected, washed, and cut into thin cross sections by hand. The slides were observed under an AxioScope A1 fluorescent microscope with filter Set 43 or a Rhodamine filter for red and green fluorescence (Ex: BP 545/25, Em: BP 525/50) (Carl Zeiss Microscopy GmbH, Göttingen, Germany) coupled to a Zeiss Axio Cam ICc1.

At the cellular level, the leaves were collected from the wild-type control and *VvmybA1* transgenic ‘Hamlin’ trees for protoplast isolation. The leaves were surface sterilized in 0.3% sodium hypochlorite solution for 5 min, rinsed four times in sterile deionized water, and chopped into small pieces. The cell walls were digested according to [29] using 0.75% (*w*/*v*) Cellulase Onozuka RS (Yakult Honsha, Tokyo, Japan), 0.75% (*w*/*v*) Macerozyme R-10 (Yakult Honsha, Tokyo, Japan), pH 5.6, at 25 ± 1.0 °C for 15 h. Protoplasts were collected in a sucrose-mannitol gradient and washed twice in BH3 media. The protoplasts were scanned under brightfield microscopy and fluorescence using the Zeiss Scope A1 fluorescence microscope.

### 2.4. Leaf Gas Exchange and Chlorophyll Fluorescence Measurements

Leaf gas exchange variables were measured on fully expanded leaves of ‘Hamlin’ trees (five trees per phenotype and three leaves per tree). The leaf gas exchange characteristics were measured using a portable infrared gas exchange analyzer (LI-6800F, Li-Cor Inc., Lincoln, NE, USA) equipped with red-blue LED light sources and external CO_2_ injectors. The leaf cuvette conditions were set as follows: 400 µmol‧mol^−1^ reference CO_2_, 500 mL‧min^−1^ flow rate, 60% relative humidity, 1200 µmol‧m^−2^‧s^−1^ PAR (photosynthetically active radiation), and 26 °C temperature.

The chlorophyll fluorescence measurements were recorded on intact dark-adapted leaves (using small, lightweight plastic leaf clips placed on the leaves overnight for 16 h) using a field portable OS-30P+ chlorophyll fluorimeter (Opti-Sciences, Inc., Hudson, NH, USA), applying dark adaptation protocols and the JIP test. Fluorescence intensities were measured under exposure to a high actinic light intensity saturation flash of 3500 µmol‧m^−2^‧s^−1^ for 3 s (to reduce and close all available PSII reaction centers). Several PSII efficiency-related variables were calculated according to Stirbet [30], Table S1.

### 2.5. Leaf Pigment Extraction

Leaf pigments (chlorophylls, carotenoids, and xanthophylls) were extracted as described by Killiny and Nehela [31]. Fully expanded leaves were sampled, frozen, and ground in liquid nitrogen. Briefly, 0.1 g of frozen leaf powder was mixed with 400 µL of 80% acetone and 240 µL of ethyl acetate and vortexed twice every 10 min. A total of 280 µL of water was added to the mixture before being centrifuged for 5 min at 8500× *g* and 4 °C. The supernatant was collected and dried under a nitrogen stream. Subsequently, dried pigments were resuspended in 200 µL of ethyl acetate, and extracts were stored at −20 °C for further analyses.

### 2.6. Analyses of Pigments with High-Performance Liquid Chromatography (HPLC)

Leaf pigments were analyzed by HPLC following Wei et al. [32] by using an Agilent 1200 system equipped with a photodiode array detector (Agilent Technologies, Santa Clara, CA, USA). Pigments were separated along a C30-YMC carotenoid column (250 × 4.6 mm i.d., 5 µm, YMC America, Allentown, PA, USA). The mobile phase composition consisted of 80% methanol, 5% methyl tert-butyl ether (MTBE), and 5% water, using the gradient profile described previously by Killiny and Nehela [31]. The column temperature was set at 25 °C, the mobile phase flow rate was fixed at 1 mL‧min^−1^, and the injection volume was 20 µL. The absorbance of pigments and peak responses were recorded at multiple spectral wavelengths (230, 278, 350, 430, and 486 nm). Chromatographic data and UV-visible spectra were analyzed using ChemStation software (versions B.03.02; Agilent Technologies). Compounds were identified by comparing experimental retention times and UV-visible spectra with that of authentic standards for chlorophyll *a*, chlorophyll *b*, lutein, and β-carotene and published data (Table S2). Calibration curves were created to calculate the pigment content from absorbance peak areas using known concentrations of the authentic standards for chlorophyll *a*, chlorophyll *b*, and lutein (Sigma-Aldrich, St-Louis, MO, USA). The calibration curve of chlorophyll a was used to calculate the content of pheophytine a and chlorophyllide a. The calibration curve of lutein was used to calculate the content of xanthophyll and carotenoid pigments (neoxanthin, antheraxanthin, trans-violaxanthin, cis-violaxanthin, zeaxanthin, and α-cryptoxanthin). Each phenotype was analyzed as five biological replicates, three leaves per biological replicate, with two technical replicates, (*n* = 30).

### 2.7. Statistical Analysis

An analysis of variance (One-way ANOVA) and a student t-test were performed using JMP Pro version 16 (SAS Institute, Cary, NC, USA). Mean separations were evaluated by a student *t*-test at a statistical significance level of *p* < 0.05.

## 3. Results

### 3.1. Generation of the Vitis vinifera mybA1 (VvmybA1) Transgenic ‘Hamlin’ Plants

**Figure 1:**
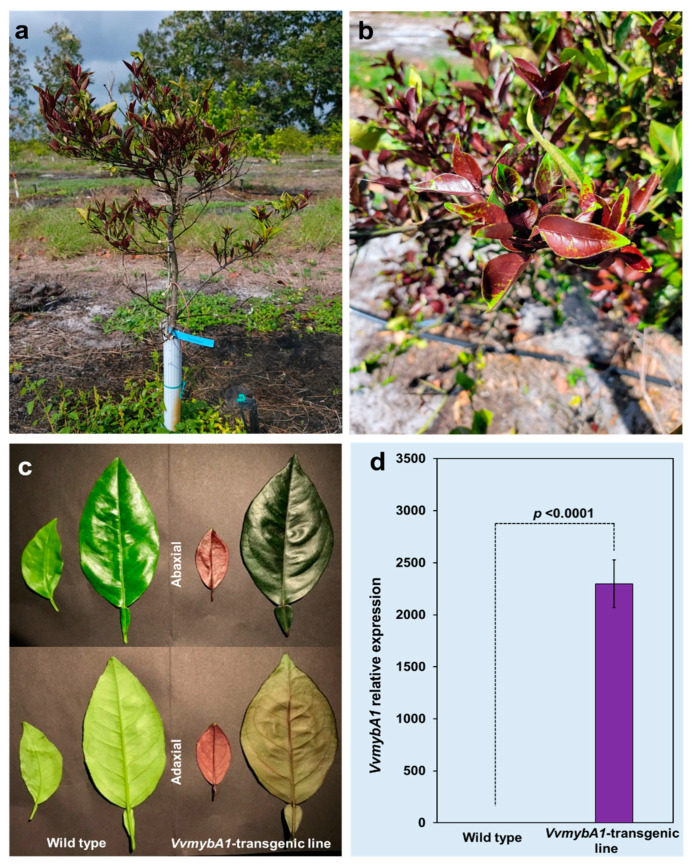
Leaf coloration and transgene expression in *VvymbA1* transgenic ‘Hamlin’ compared with the wild-type control (**a**,**b**) trees planted in the field. (**c**) Phenotype differences in the abaxial and adaxial epidermis of juvenile and mature leaves in both control and transgenic plants. (**d**) Relative gene expression of *VvmybA1* using real-time PCR.

We characterized a putative *VvmybA1* transcription factor gene as described in our previous study [12]. In this study, a population of transgenic ‘Hamlin’ sweet orange lines expressing the *VvmybA1* gene was obtained. A transgenic line was identified based on the visual anthocyanin expression in the leaves, which was subsequently used in this study (Figure 1). Clones of this line that were planted in the field also exhibited a high anthocyanin expression, indicating that the *VvmybA1* transcription factor gene stably expressed even under field conditions (Figure 1a,b). The morphological appearance of *VvmybA1* leaves indicated that anthocyanin pigments are visualized in both the adaxial and abaxial epidermis of juvenile leaves. However, mature leaves visually had less anthocyanin production compared with juvenile leaves, and anthocyanin production was higher than control non-transgenic ‘Hamlin’ leaves (Figure 1c). The expression of *VvmybA1* was further verified by quantitative PCR (qPCR), and the transcript level of *VvmybA1* was significantly expressed compared to the control ‘Hamlin’ trees (Figure 1d).

### 3.2. Localization of Anthocyanins in Leaf Tissues

**Figure 2:**
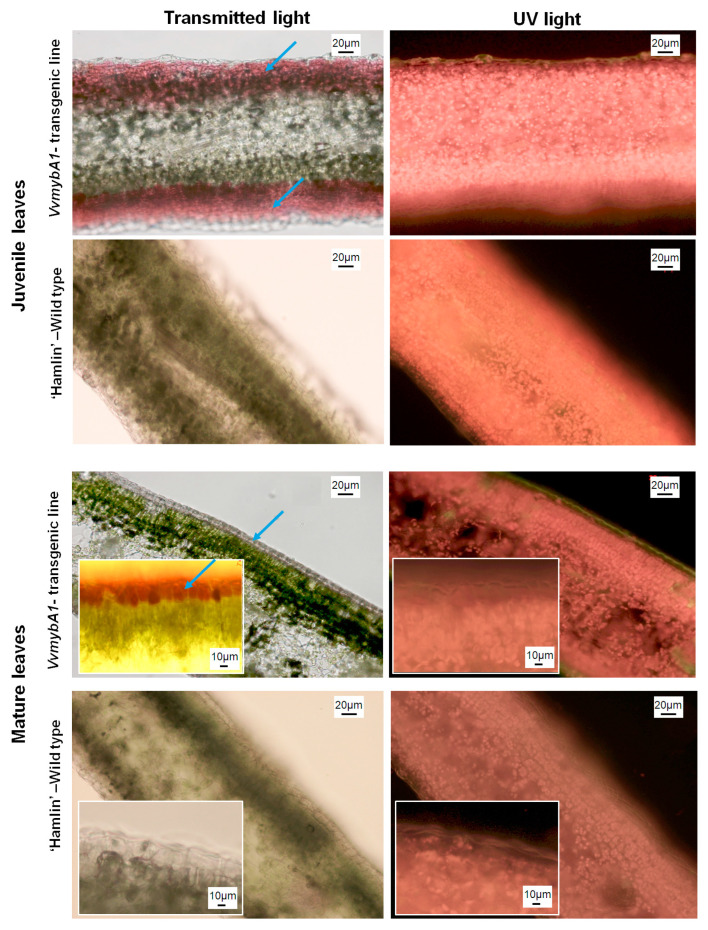
Microscopic imaging of cross sections of juvenile and mature leaves of control ‘Hamlin’ VvymbA1 transgenic ‘Hamlin’ as visualized under transmitted light and UV light. The images were visualized under fluorescence to show the chloroplasts in the leaves. Note that anthocyanin pigments are localized in the mesophyll of juvenile leaves and are restricted to the epidermal cells in mature leaves. The blue arrows indicate the positions of anthocyanin accumulation.

The cross-sectioning of juvenile and mature leaves revealed the presence of anthocyanin pigments in leaf tissues. Juvenile leaves exhibited an accumulation of pigment on the mesophyll cells, while sections of mature leaves showed that accumulations of anthocyanin are limited to epidermal cells. In mature leaves, mesophyll cells that contained tightly packed elongated cells with a vast number of green lens-shaped chloroplasts did not contain the anthocyanin pigment. By visualizing chloroplasts in both control and anthocyanin-expressing leaves, fluorescence light microscopy showed no differences in the number of chloroplasts (Figure 2).

**Figure 3:**
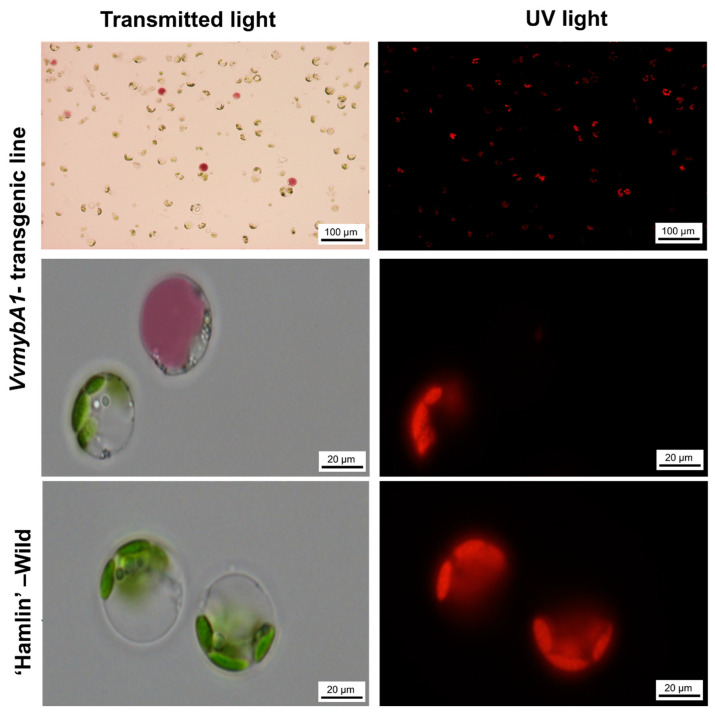
Isolated protoplasts from leaves of the *VvmybA1* transgenic line and ‘Hamlin’ wild type. Left images were visualized under transmitted light showing the accumulation of anthocyanin in the vacuoles. The right images were visualized under fluorescence to show the chloroplasts’ presence in the protoplasts.

At the cellular level, the isolated protoplasts from the transgenic juvenile leaves showed a mixture of anthocyanin-enriched cells with small-sized chloroplasts (Figure 3), and most of the cells contained only chloroplasts. The anthocyanin accumulated in the vacuoles (Figure 3).

### 3.3. Expression of VvmybA1 Does Not Alter Leaf Gas Exchange

**Figure 4:**
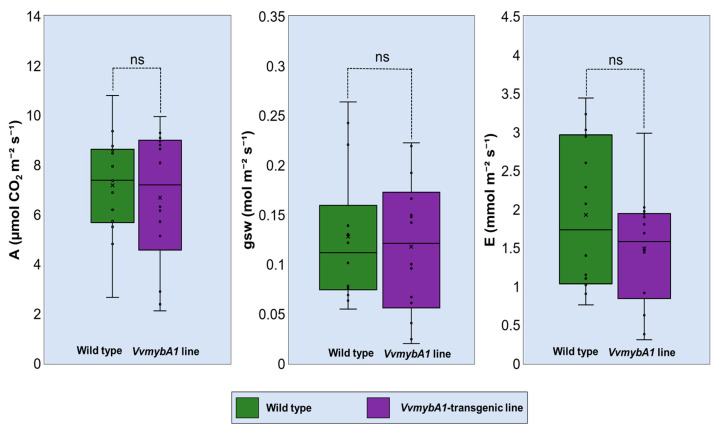
Gas exchange parameters include the net assimilation of CO_2_ (A), transpiration rate (E), and stomatal conductance to water vapor (gsw) in the leaves of *VvmybA1* transgenic ’Hamlin’ compared with the wild-type control. ns—No significance. Individual data points falling outside the whiskers in box plot, are indicated by circles or "x" marks.

To determine whether the expression of the *VvmybA1* transcription factor and the production of anthocyanin alter the gas exchange in Citrus sinensis ‘Hamlin’ sweet orange, we measured the net rate of CO_2_ assimilation (ACO_2_), the stomatal conductance (gsw), and the transpiration rate (E). ‘Hamlin’ leaves with anthocyanin accumulation because of the expression of the *VvmybA1* gene showed no significant differences in ACO_2_ when compared to control wild leaves. Both phenotypes showed values of ACO_2_ in the average range of sweet orange (*Citrus sinensis*) leaves (6.0–7.0 µmole CO_2_‧m^−2^‧s^−1^). Similarly, stomatal conductance showed similar performance in both *VvmybA1* and control leaves (0.1–0.15 mol‧m^−2^‧s^−1^). We recorded a slight reduction in the transpiration rate (E) in the transgenic lines in comparison to the wild-type control, but it was not statistically different (Figure 4).

### 3.4. Expression of VvmybA1 Gene Causes Differential Response of Photosynthetic Machinery

**Figure 5:**
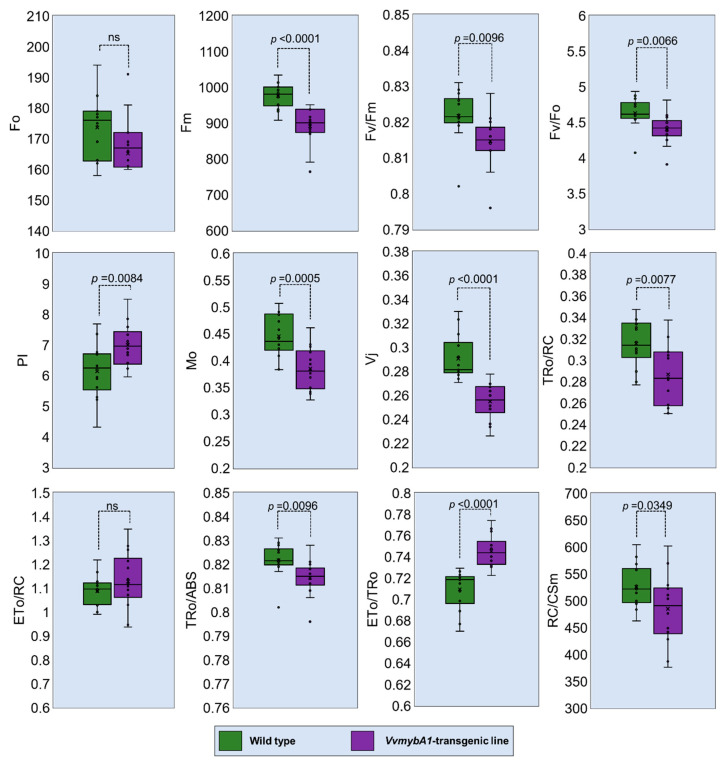
Chlorophyll fluorescence measurements (Fo, Fm, Fv/Fm, Fv/Fo, PI, Mo, Vj, TRo/RC, ETo/RC, TRo/ABS, ETo/TRo, and RC/CSm) in the leaves of *VvmybA1* transgenic ‘Hamlin’ compared with wild-type control. ns—No significance.

Chlorophyll fluorescence analysis showed differential responses between the two phenotypes (Figure 5). There was a significant reduction in Fv/Fo and PSII maximum quantum yield (Fv/Fm or φPo) values in *VvmybA1* transgenic leaves compared to control (0.821 ± 0.006 for control leaves and 0.817 ± 0.007 for *VvmybA1* leaves, *p* = 0.0096). Although there is a decrease in the values of φPo of *VvmybA1* leaves, it still ranges within the normal average of C3 plant leaf values. Concerning the performance indices, we recorded higher performance index per absorbance (PIabs) values in the transgenic line compared with wild type (Table 2), but no difference in PItot, which is the performance index from absorbance through reduction in the PSI receptors. Looking at the components of PI, in addition to φPo, the wild type had greater ∂_RE1_, while the *VvmybA1* transgenic plants had greater ψ_ET1_, and there were no differences in γ_RC_. When looking at specific fluxes, the *VvmybA1* transgenic plants had lower fluxes per cross section at all stages, from absorbance through J^RE^; however, the higher efficiency of transfer from Qa to Qb (ψ_ET1_) led to a much smaller magnitude of difference between the transgenic line and the wild type at J^ET^, but the increased ∂_RE1_ led to a greater increase in the final flux through the PSI in the wild type than in the *VvmybA1* transgenic plants.

### 3.5. Quantifications of Chlorophyll and Carotenoid Pigments

**Figure 6:**
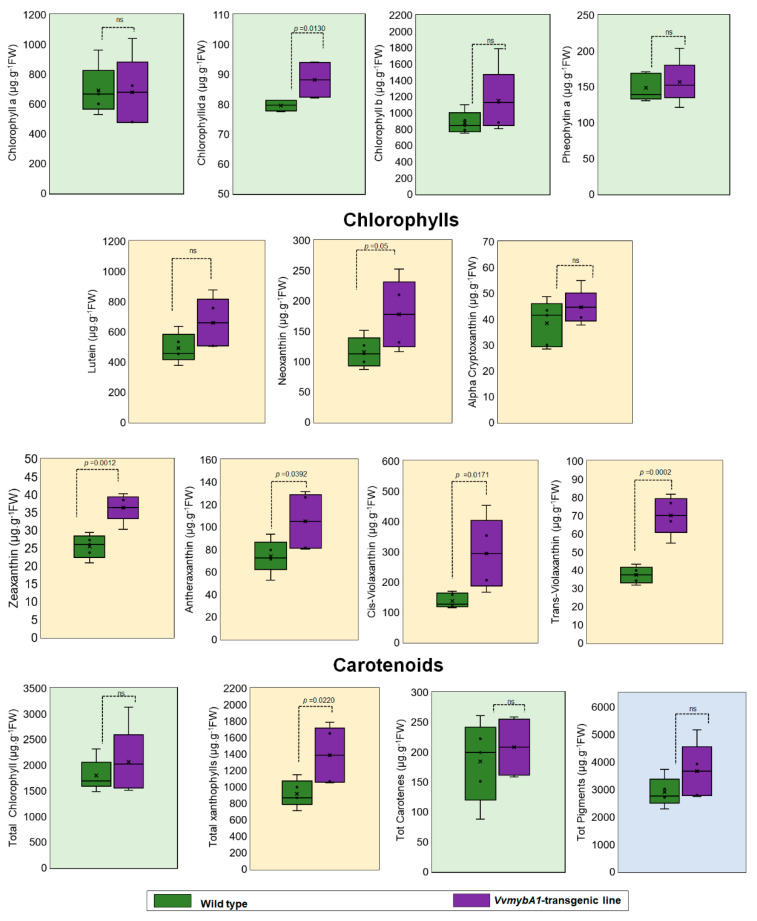
Changes in the content of chlorophylls (chlorophyll a, chlorophyllide a, chlorophyll b, and pheophytin a, total chlorophylls) and carotenoids (lutein, neoxanthin, trans-violaxanthin, cis-violaxanthin, antheraxanthin, zeaxanthin, and α-cryptoxanthin) in the leaves of VvmybA1 transgenic ‘Hamlin’ compared with wild-type control. ns—No significance.

The results obtained from chlorophyll fluorescence analysis suggest that anthocyanin production may affect the other foliar pigments upon expression of the *VvmybA1* gene. To verify this assumption, the total pigment, total chlorophyll, total xanthophyll, and total carotene contents were estimated in the two phenotypes. Of the chlorophyll pigments, only chlorophyllide a showed a significant increase (*p* = 0.0130) in the transgenic line compared with the wild type, recording 79.508 ± 1.792 and 88.142 ± 5.798, respectively, Figure 6. However, as the chlorophyll a (Chl *a*) content showed a non-significant trend toward higher values in green leaves and the chlorophyll b (Chl *b*) content showed a non-significant trend toward higher content in *VvmybA1* leaves, the chlorophyll a to chlorophyll b ratios were significantly higher in the control leaves (0.79 ± 0.06) when compared to the *VvmybA1* leaves (0.58 ± 0.03) (Figure 6). Moreover, there were no significant differences in the carotenoid content in the two phenotypes (Figure 6).

Regarding the individual xanthophylls and carotenes pigments (Figure 6), we observed a significant induction in xanthophyll pigments upon the expression of the *VvmybA1* gene. The content of zeaxanthin, trans-violaxanthin, trans-violaxanthin, antheraxanthin, and total xanthophylls were significantly higher in the leaves expressing anthocyanin compared to the control leaves (Figure 6). Therefore, the ratio of xanthophyll to total chlorophyll contents (Table 2) was significantly higher in *VvmybA1* leaves when compared to the control leaves. In contrast, the ratio of Chl *a*/Chl *b* and zeaxanthin/violaxanthin contents were significantly higher in the control leaves compared with the transgenic leaves. The carotenes/total chlorophylls ratio expressed no significant differences when the *VvmbA1* transgenic and control leaves were compared (Table 3).

### 3.6. Expression of VvmybA1 Gene Induces Higher Expression of RuBP and RCA

**Figure 7:**
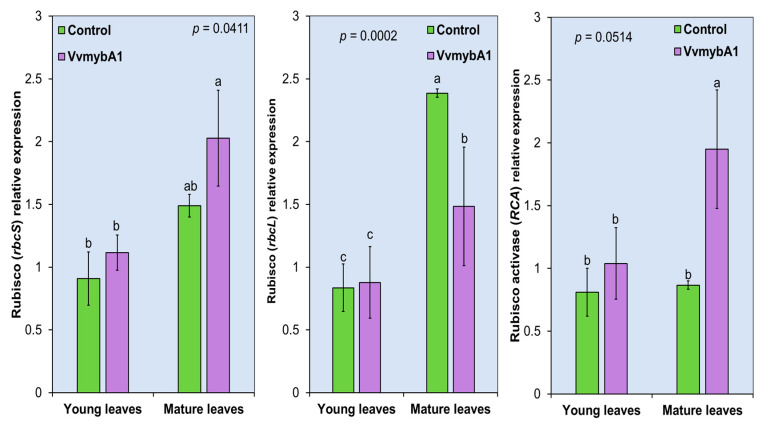
Relative expression of Rubisco transcripts using real-time PCR; *rbcL*: Ribulose-1,5-bisphosphate carboxylase/oxygenase large subunit; *rbcS*: small subunit of Rubisco; and *RCA*: Rubisco activase in the leaves of *VvmybA1* transgenic ‘Hamlin’ compared with wild-type control. Different letters (a, b, and c) indicate significant differences.

We observed variation in the expression of *RuBP* and *RCA* in the comparison between the young and mature leaves in both control and transgenic ‘Hamlin’ plants. The relative expression is significantly higher in the mature leaves than in the young leaves (Figure 7). Despite the absence of significant changes in gas exchange rates, a substantial relative expression of *RCA* was documented in the mature leaves of transgenic plants at the optimal temperature. These observations suggest an activated state of RuBP in anthocyanin-rich mature leaves. Moreover, the activated state of Rubisco in these mature leaves maintained a heightened expression of the ribulose-1,5-bisphosphate carboxylase/oxygenase large subunit (*rbcL*). In contrast, the mature leaves of the control plants exhibited a higher relative expression of the small subunit of Rubisco (*rbcS*) compared to the transgenic plants.

## 4. Discussion

The allocation of anthocyanins in plant tissues is genetically controlled by the tissue-specific expression of regulatory genes [33]. Anthocyanins accumulate in peripheral layers, especially in the upper epidermis when subjected to direct light [34]. In the expanding leaves of mango and cacao, red pigmentation was observed in the abaxial surfaces [35]. Abaxial leaf surfaces exhibit systematically more visible reflectance and light sensitivity compared with the adaxial surfaces, whatever the species [36]. In some species, they exhibit throughout the leaf in mesophyll tissue as well as trichomes [37,38]. Heterogeneity in cell response to stimuli leads to a gradual increase in pigmentation intensity in the plant tissues [39].

In the current study, a genetically modified ‘Hamlin’ sweet orange expressing anthocyanins was engineered, and the photosynthetic performance was studied and compared with the wild-type control plants under optimal growth conditions. The transgenic ‘Hamlin’ plants exhibit an intense purple color in the juvenile leaves compared to the mature leaves. It accumulated in the abaxial surface of the epidermal tissues. At the cellular level, we confirmed the localization of anthocyanin pigments in the epidermal cells of mature leaves and in the mesophyll tissue and that the pigments typically accumulate in the vacuoles of plant cells of juvenile leaves of *VvmybA1* transgenic ‘Hamlin’.

Under the optimum growth conditions of citrus trees, we recorded no significant differences in leaf gas exchange parameters, indicating normal growth and photosynthesis in both phenotypes. This finding is consistent with the performance of sweet basil plants [40] and the leaf morphotypes of *Tipularia discolor* [41]. Differential changes in gas exchange variables induced by anthocyanin biosynthesis during different ages of leaves [42] or under environmental stress [43] were reported, but these results were not reflected in the present study.

In relation to chlorophyll fluorescence measurements, there is a noticeable yet marginal reduction in the Fv/Fm ratio observed in the transgenic line when compared to the wild type. However, this decrease is not substantial enough to imply photodamage to PSII. Lysenko et al. [44] demonstrated that variations in the Fv/Fm ratio can be influenced by factors such as the color of the measuring light or the saturating pulse, potentially explaining the slight decrease observed in the mutant. Furthermore, the OJIP analysis presents several variables but remains a topic of controversy, as highlighted by Maxwell and Johnson [45]. Specifically, the increase in OJIP may not accurately represent the biphasic nature of QA photoreduction. This rise in JIP might be associated with a “thermal phase,” potentially originating from either the reduction in the PQ pool or conformational changes within the reaction center of PSII.

Additionally, our analysis of the biophysical OJIP variables calculated from the transient rise of chlorophyll fluorescence indicates that, in VvmybA1 leaves, the photosynthetic machinery adapts to reduced irradiance in a mesophyll partly shielded by additional anthocyanins. The slight decrease in ΦPo results from a reduction in Fm, which was unexpected, as other species typically exhibit higher Fm in shade acclimation to increase absorbance [46]. The lack of absorbance-enhancing acclimation is supported by the unchanged γRC, which is related to antenna size. However, antenna size may often be optimized for low irradiance conditions, as argued by Wu et al. [47], and citrus likely falls into the category of plants that do not fully acclimate to high irradiance conditions [48].

The high efficiency of transfer from Qb to PSI and higher dissipation fluxes in wild-type plants indicate acclimation to divert excess energy away from PSII, a mechanism that may not be necessary in trees with a protective layer of anthocyanin. Nevertheless, *VvmybA1* leaves acclimate through increased efficiency of transfer from Qa to Qb to achieve the same PItot as wild-type leaves. This suggests that these trees may perform better under high-irradiance and high-temperature conditions, conditions that typically reduce productivity as they require greater investment in photoprotective mechanisms [49,50]. Moreover, the efficiency/probability with which a PSII-trapped electron is transferred from QA to QB (as measured by ΨET20) was higher in VvmybA1 leaves, possibly compensating for the reduction in photon absorption.

We quantitatively reported a similar content of chlorophyll pigments in the transgenic and control leaves. These findings are consistent with our previous study on *VvmybA1* transgenic juvenile leaves of ‘Mexican lime’ *Citrus aurantifolia* [17] and *Arabidopsis thaliana* mutant PAP1-D overexpressing anthocyanin [19]. Similarly, natural anthocyanin accumulation in cyanic leaves leads to insignificant changes in total chlorophyll contents. On the other hand, a reduction in the chlorophyll *a* to chlorophyll *b* ratio was reported in several species including *Prunus cerasifera* [51], *Quercus coccifera* [52], *Oxalis triangularis* [53], and *Pelargonium × hortorum* [54]. The incident decline in the chlorophyll *a* to chlorophyll *b* ratio observed may be due to a shared pattern with shade leaves that display the “shade acclimation or avoidance syndrome.” Anthocyanin accumulation in the adaxial and abaxial epidermal cells of *VvmybA1* transgenic leaves creates a kind of sunscreen, imposing shade on the subjacent mesophyll cells and reducing light intensity. Shaded leaves that receive less light intensity and a higher proportion of far-red and green light relative to red and blue light (because the upper leaves absorb mainly blue and red wavelengths) try to maximize light capture through the enlargement of light-harvesting complexes (LHCs) of photosystems II and I (PSII and PSI) and the production of more peripheral LHCs per reaction center [55]. Because LHCs have a higher proportion of chlorophyll *b* than other complexes, this can explain why shade leaves show a decreasing gradient of chlorophyll a to chlorophyll b from the top to the bottom of the canopy [56].

Xanthophyll and carotenoid pigments play crucial roles in photoprotection from excessive energy absorbed by leaves [57]. They also participate in light harvesting within the blue and green regions, passing energy to chlorophyll [58]. The capacity of *VvmybA1* transgenic leaves to adjust xanthophyll pigment content to maximize light absorption confirms the function of anthocyanins in maintaining photosynthetic machinery efficiency and a less reduced state in the chloroplast, indicating less reactive oxygen species activity. The xanthophyll cycle regulates NPQ and modulates the kinetics of formation and relaxation [57]. During times of excess light exposure, reversible conversion takes place to induce zeaxanthin formation via the enzyme violaxanthin de-epoxidase, whereas zeaxanthin de-epoxidase is necessary to convert zeaxanthin to violaxanthin by de-epoxidation of violaxanthin [57]. Accumulation of zeaxanthin kinetically correlates with the formation and long-term relaxation scale (qI). Zeaxanthin also has crucial antioxidant properties that protect membrane lipids from peroxidation [59]. In *Arabidopsis*, the xanthophyll cycle pool size is enlarged upon overexpression of the gene encoding β-carotene hydroxylase [60]. The increased zeaxanthin concentration following activation of the xanthophyll cycle enhanced plant resistance under oxidative stress due to the anti-oxidative characteristics of carotenoids [59,60].

The anthocyanin-rich mature ‘Hamlin’ leaves in our study show higher relative expression of the Rubisco gene than the control leaves. These findings correlated with an inhibition of the PSII yield, a response that occurs by light exposure followed by activation of the xanthophyll cycle. The photosynthetic rate is limited by the activity of Ribulose-bisphosphate carboxylase (E.C. 4.1.1.39) by the electron-transport capacity of the chloroplast to regenerate it. Suganami et al. [61] produced transgenic rice (*Oryza sativa*) plants co-overexpressing *RubP* and *RCA*. The overexpression of *rbcS* and *RCA* showed an increase in the rate of CO_2_ assimilation in the transgenic plants at moderately elevated temperatures within the optimal temperature range. However, the overproduction of Rubisco is accompanied by a decline in Rubisco activation [62]. This decline may refer to a suppression in the activation state of Rubisco that hinders the improvement of photosynthesis in crops [61]. These findings suggest that *RuBP* and *RCA* are necessary to improve photosynthesis and crop productivity.

## 5. Conclusions

The expression of the *VvmybA1* gene results in enhanced permanent anthocyanin production in ‘Hamlin’ sweet orange leaves. To compensate for light attenuation caused by anthocyanins, the leaves simultaneously enhance light capture through increased carotenoid production. Mature leaves enriched with anthocyanins exhibit higher relative expression of the *RuBP* gene compared to control leaves. Furthermore, there is a slight inhibition of PSII yield—a response that may be initiated by light exposure followed by the activation of the xanthophyll cycle or changes in the absorption of the measuring light. This process confirms efficient photoacclimation of the photosynthetic apparatus, allowing the transgenic line to maintain photosynthetic performance similar to that of the wild type. Notably, the impact of anthocyanin expression resembles the effects of treatments with red clay or shade, but it offers a sustainable effect. Further investigations could provide additional details about the photoprotective role of anthocyanin-producing ‘Hamlin’ sweet orange under various environmental stressors induced by a changing climate.

## Figures and Tables

**Table 1:** List of the primer sequences used in SYBR Green-based real-time PCR assay.

Accession Number	Gene Symbol	Primer Sequences (5′ to 3′)
AB097923	*VvmybA1*	*F*-TGGCATAGTCACCACTTCAAA
		*R*-CTTGTGAGGGTGAGGCTTTAT
XM_052431503.1	*rbcL*	*F*-GTTGGAGGGACCGTTTCTTAT
		*R*-CCCTGCAGTAGCATTCAAGTA
XM_006482191.4	*rbcS*	*F*-CGGAAGAGTCCAATGCATGA
		*R*-AGCGGAGAAGGTAACTGA
XM_006480794.4	*RCA*	*F*-CTTTGCCAAGATGGGAATCAAC
		*R*-TGTCTGCTGCTTCACGATAC
XM_006464503.3	*β-actin*	*F*-GCTGCCTGATGGCCAGATC
		*R*-AGTTGTAGGTAGTCTCATGAA

**Table 2:** Selected variables calculated from chlorophyll a, fluorescence transients (JIP) for wild type, and *VvmbA1* transgenic ‘Hamlin’ sweet orange (*Citrus* x *sinensis*).

	Variable	Anthocyanin		Wild type		*p*-Value
		Mean ±	Std. Err.	Mean ±	Std. Err.	
Performance indices	PI_abs_	7.0	0.2	6.1	0.2	0.008
	PI_tot_	4.4	0.2	4.3	0.3	0.681
Components of PI	φ_Pο_	0.815	0.002	0.822	0.002	0.008
	ψ_ΕΤ1_	0.746	0.004	0.71	0.005	<0.0001
	∂_RE1_	0.385	0.008	0.406	0.008	0.076
	γ_RC_	0.35	0.006	0.35	0.003	0.997
Phenomenological fluxes	J^ABS^	892	14	976	9	<0.0001
	J^DI^	165	4	174	3	0.076
	J^TR^	727	11	803	7	<0.0001
	J^ET^	541	7	569	6	0.003
	J^RE^	255	6	281	6	0.005

**Table 3:** The ratio of leaf pigments detected in the control and *VvmybA1* leaves.

Pigment Ratios	Control	*VvmybA1*	*p*-Value
Chlorophyll *a*/Chlorophyll *b*	0.795 ± 0.078 *	0.590 ± 0.036	0.0007 *
VAZ/Total chlorophylls	0.154 ± 0.011	0.259 ± 0.055	0.0031 *
Lutein/Total chlorophylls	0.269 ± 0.013	0.329 ± 0.036	0.0089 *
Neoxanthin/Total chlorophylls	0.063 ± 0.004	0.086 ± 0.011	0.0028*
Xanthophylls/Total chlorophylls	0.509 ± 0.026	0.699 ± 0.101	0.0037 *
Carotenes/Total chlorophylls	0.100 ± 0.026	0.106 ± 0.018	ns
Total chlorophylls	0.609 ± 0.049	0.805 ± 0.120	0.0097 *
Zeaxanthin/Violaxanthins	0.151 ± 0.005	0.111 ± 0.037	0.0471

* Numbers represent means of five replicates ± standard deviation, ns—No significance.

## Data Availability

Data generated or analyzed during the current study are included in this published article and are available from the corresponding author on reasonable request.

## Supplementary Materials

The following supporting information can be downloaded at: https://www.mdpi.com/article/10.3390/plants12233965/s1. Table S1: Calculation and explanation of selected variables calculated from chlorophyll a fluorescence transients (JIP test); Table S2: Chromatographic and spectral characteristics of different pigments identified in citrus leaves using HPLC.
