# Supplementary material for: Influence of Anthocyanin Expression on the Performance of Photosynthesis in Sweet Orange, Citrus sinensis (L.) Osbeck
Source: Plants (Basel). 2023 Nov 24;12(23):3965. doi: 10.3390/plants12233965 (PMC10708278; doi:10.3390/plants12233965)
Supplement: Supplementary file 1 [file plants-12-03965-s001.zip › plants-2653837-supplementary.pdf]

Supplementary Table S1. Calculation and explanation of selected variables calculated from chlorophyll a fluorescence transients (JIP test).

**Parameter**	**Formula**	**Meaning**
F_v/F_m or φ_{Po}	$(F_m - F_o)/F_m$	Maximum quantum efficiency of PSII
γ_{RC}	$\frac{Chl_{RC}}{Chl_{Total}} = \frac{RC/J^{ABS}}{1 + RC/J^{ABS}} = \frac{M_o}{V_J \cdot \varphi_{Po} + M_o}$	Probability that PSII acts as a reaction center
ψ_{ET1}	$\frac{J_0^{TR}}{J^{ABs}} \cdot \frac{J^{ABS}}{CS} = \frac{F_m}{F_o} \cdot F_m$	Efficiency of electron transfer from plastoquinone a (Q_a) to Q_b
∂_{RE1}	$\frac{J_o^{RE1}}{J_o^{ET2}} = \frac{1 - V_I}{1 - V_J}$	Efficiency of electron transfer from Q_b to PSI
PI_{abs}	$\frac{\varphi_{Po}}{1 - \varphi_{Po}} \cdot \frac{\Psi_{ET1}}{1 - \Psi_{ET1}} \cdot \frac{\gamma_{RC}}{1 - \gamma_{RC}}$	Performance index of energy conservation from photons absorbed by PSII antenna until the reduction of Q_b
PI_{tot}	$\frac{\varphi_{Po}}{1 - \varphi_{Po}} \cdot \frac{\Psi_{ET1}}{1 - \Psi_{ET1}} \cdot \frac{\gamma_{RC}}{1 - \gamma_{RC}} \cdot \frac{\delta_{RE1}}{1 - \delta_{RE1}}$	Performance index of energy conservation from photons absorbed by PSII antenna until the reduction of PSI acceptors
Specific fluxes per cross-section		
J^{ABS}	$\frac{J^{ABS}}{CS} = F_m$	Flux of absorbed photons per cross section
J^{TR}	$\frac{J_0^{TR}}{J^{ABs}} \cdot \frac{J^{ABS}}{CS} = \frac{F_m}{F_o} \cdot F_m$	Maximum trapped exciton flux per cross section
J^{ET}	$\frac{J_0^{ET}}{J^{ABs}} \cdot \frac{J^{ABS}}{CS} = \varphi^{ET} \cdot F_m$	Electron transport flux from Q_a to Q_b per cross section
J^{RE}	$\frac{J_0^{RE1}}{J^{ABS}} \cdot \frac{J^{ABS}}{CS} = 1 - V_i \cdot Fm$	Electron transport flux until PSI acceptors per cross section

Supplementary Table S2. Chromatographic and spectral characteristics of different pigments identified in citrus leaves using HPLC.

Pigments	Retention time in minute	ʎmax in nm at peaks (Absorbance at UV-visible 430 nm)	Identification
***Trans*-violaxanthin**	11.4	440 and 470	Killiny and Nehela 2017
**Neoxanthin**	12.3	440 and 470	Killiny and Nehela 2017
***Cis*-violaxanthin**	13.9	440 and 470	Killiny and Nehela 2017
**Antheraxanthin**	16.4	420 and 440	Lee et al., 2001, Melendez Martinez e al., 2003, Lux et al., 2019
**Chlorophyll b**	17.9	470	Authentic standards
**Lutein**	20.5	440 and 480	Authentic standards
**Chlorophyll a**	24	440	Authentic standards
**Zeaxanthin**	29.3	435 and 475	Killiny and Nehela 2017
**α-Carotene**	39	440 and 470	Killiny and Nehela 2017
**β-Carotene**	45.4	445	Authentic standards
**α-Cryptoxanthin**	49.3	410 and 435	Killiny and Nehela 2017
**Pheophytin a**	51.2	410	Killiny and Nehela 2017

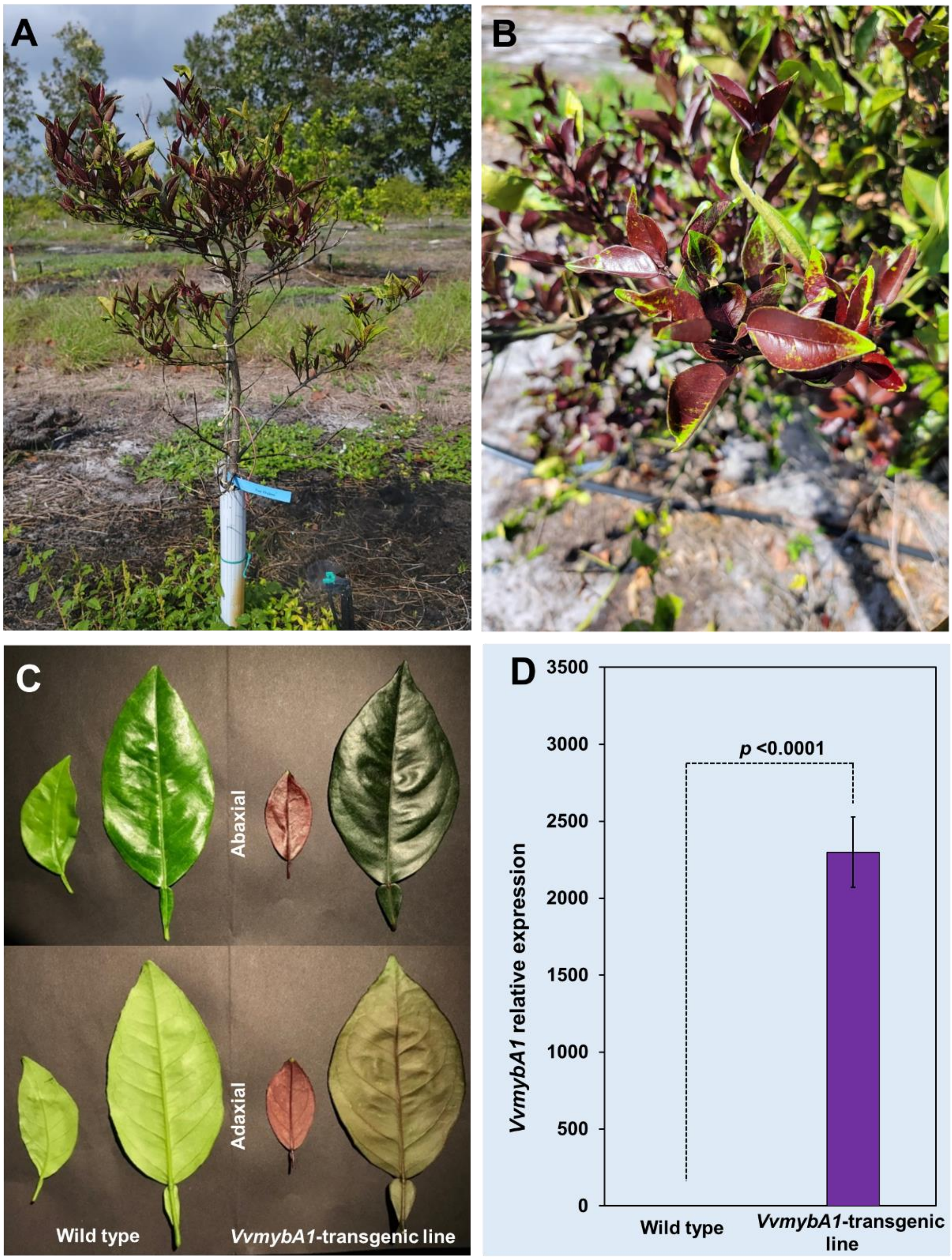

Figure S1. Leaves coloration and transgene expression in *VvymbA1* transgenic 'Hamlin' compared with the control wild type. (A-B) trees planted in the field. (C) Phenotype differences in abaxial and adaxial epidermis of juvenile and mature leaves in both control and transgenic plants (D) Relative gene expression of *VvmybA1*.

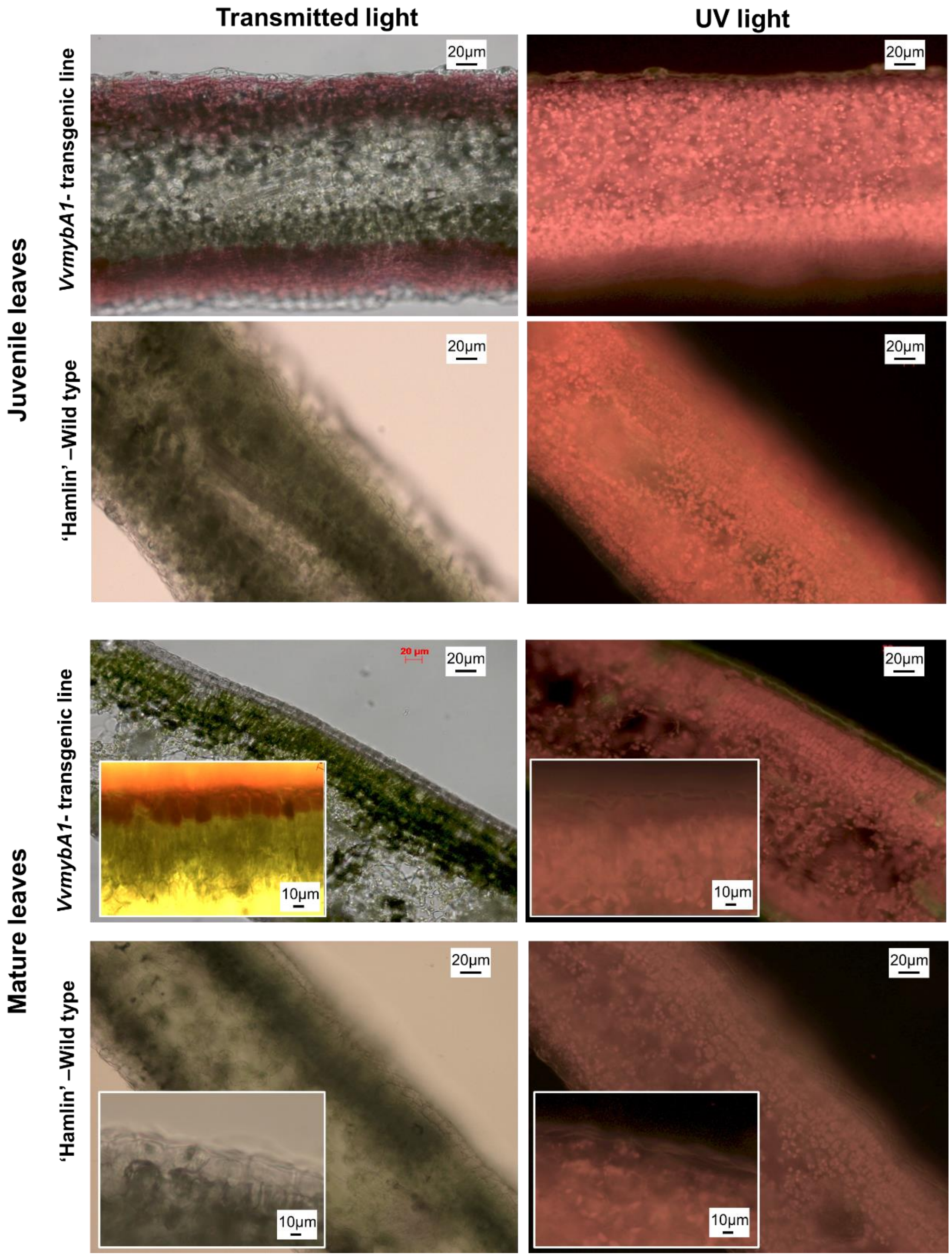

Figure S2. Microscopic imaging of cross sections of juvenile and mature leaves of control 'Hamlin' *VvymbA1* transgenic 'Hamlin' as visualized under transmitted light and UV light. The images were visualized under fluorescence showing the chloroplasts in the leaves. Note that anthocyanin pigments are localized in mesophyll of juvenile leaves while restricted to the epidermal cell in the mature leaves. represent the epidermal tissues (a-c) and represent the mesophyll tissues.

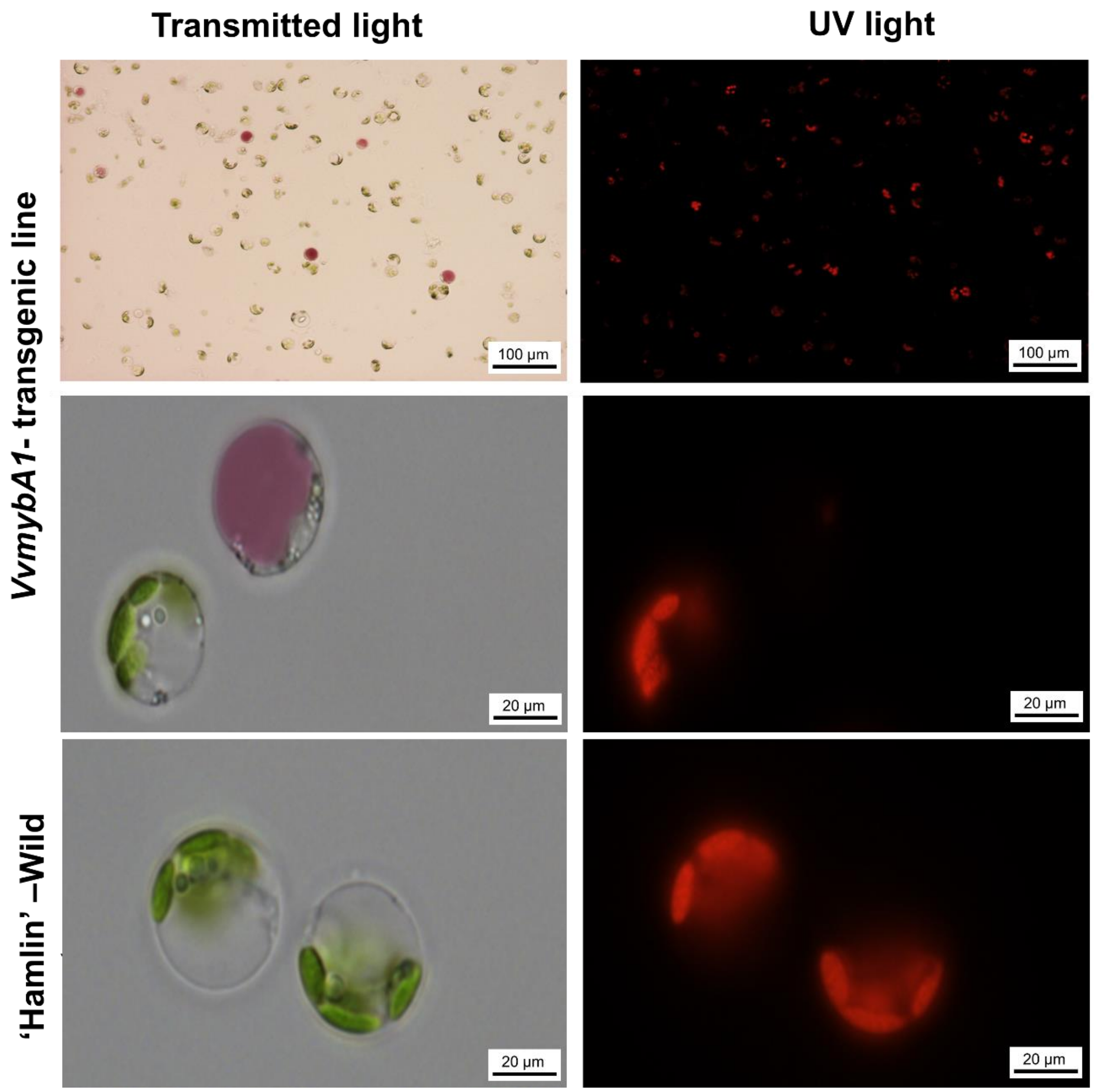

Figure S3. Isolated protoplasts from leaves of *VvmybA1*- transgenic line and 'Hamlin' wild type and Left images were visualized under transmitted light showing the accumulation of anthocyanin in the vacuoles. The right images were visualized under fluorescence showing the chloroplasts presence in the protoplasts.

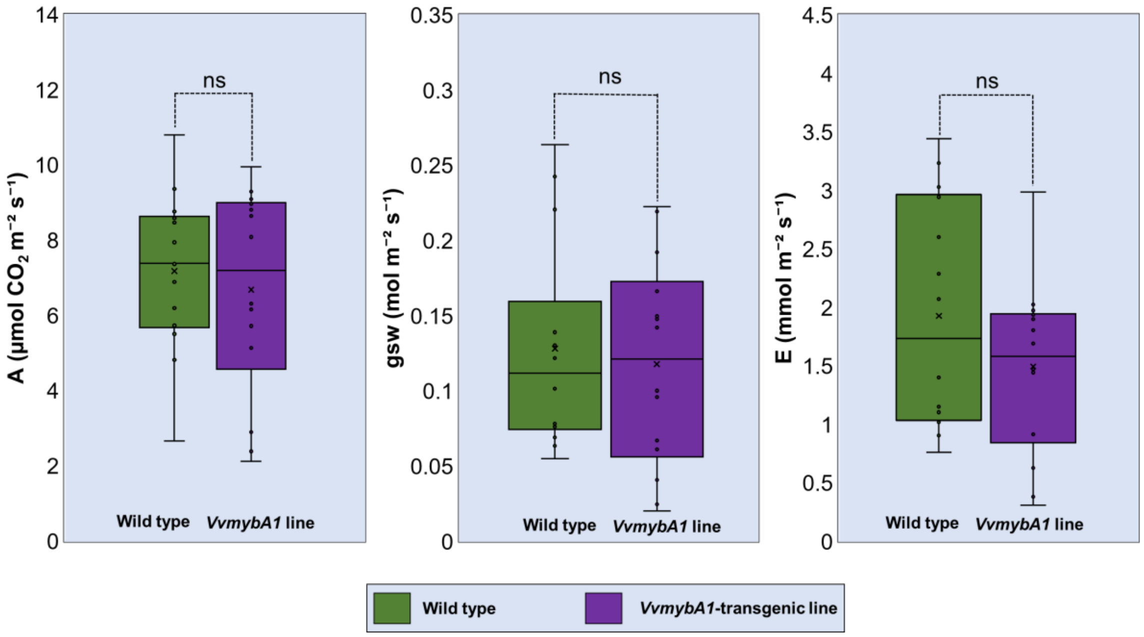

Figure S4. Gas exchange parameters include net assimilation of CO_2 (*A*), transpiration rate (*E*) and stomatal conductance to water vapor (g_{sw}) in the leaves of *VvmybA1*- transgenic 'Hamlin' compared with the control wild type.

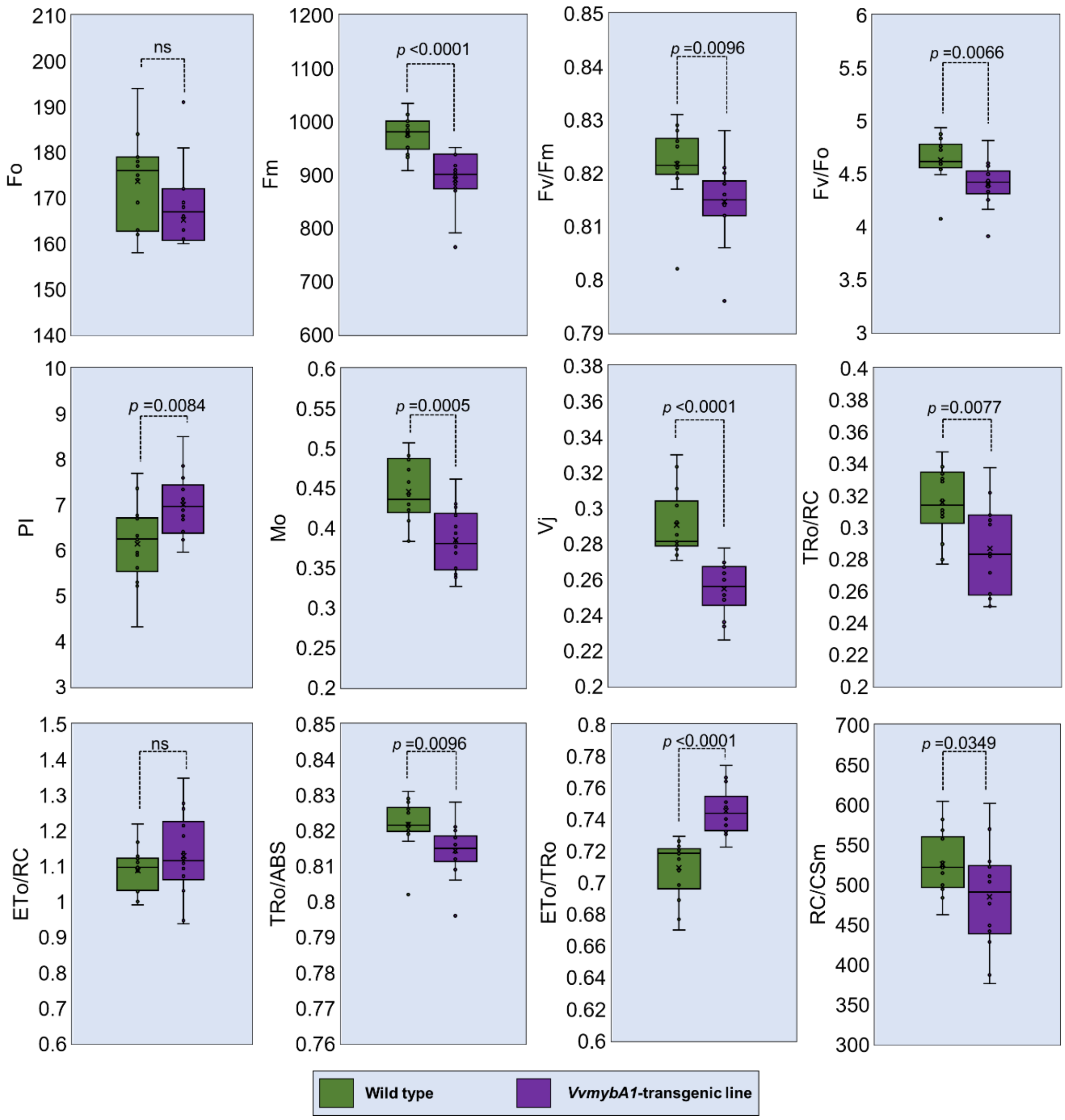

Figure S5. Chlorophyll fluorescence measurements (Fo, Fm, Fv/Fm, Fv/Fo, PI, Mo, Vj, TRo/RC, ETo/RC, TRo/ABS, ETo/TRo and RC/CSm) in the leaves of *VvmybA1* transgenic 'Hamlin' compared with control wild type.

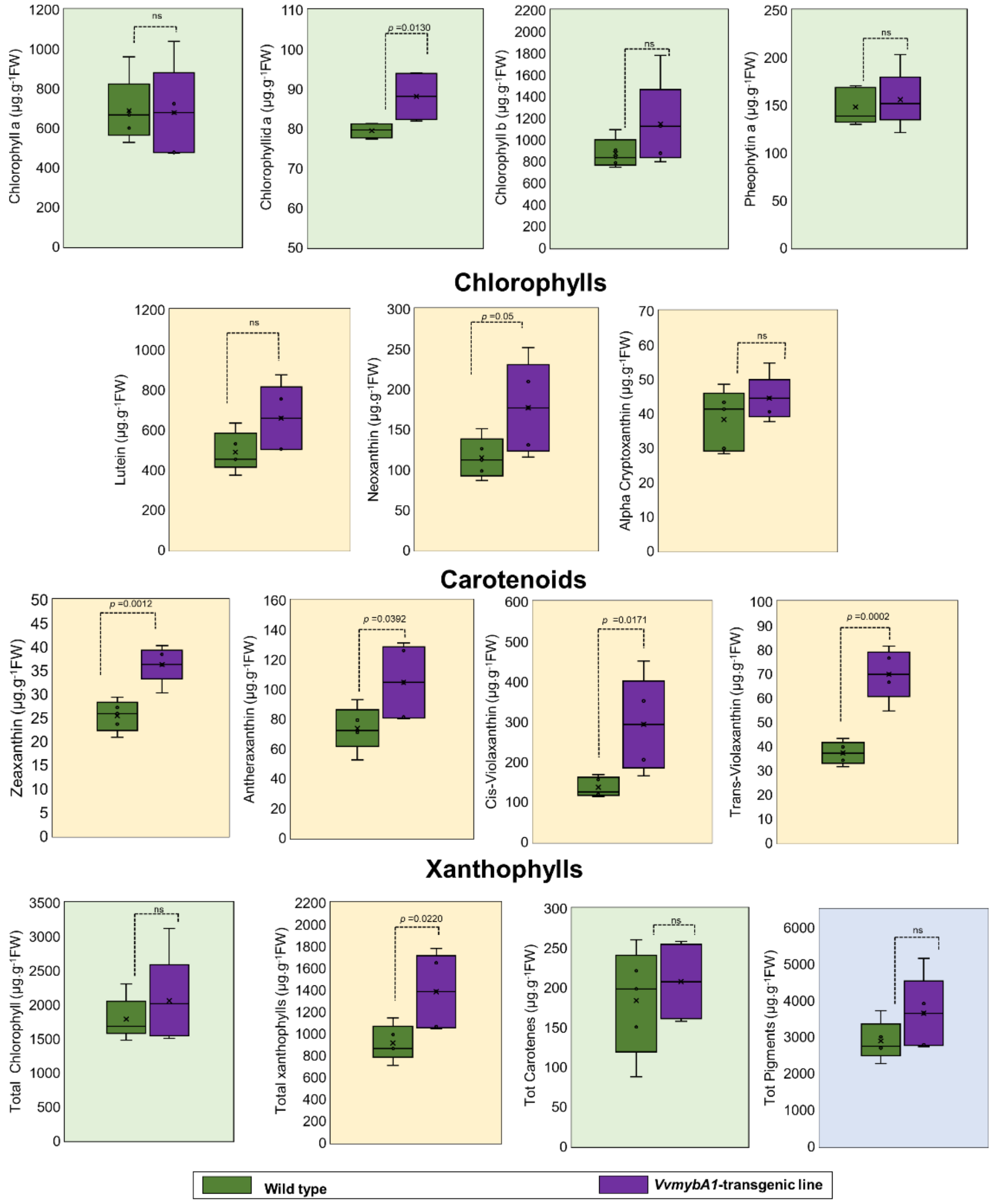

Figure S6. Changes in the content of chlorophylls (chlorophyll *a*, chlorophyllide *a*, chlorophyll *b*, and pheophytin *a*, total chlorophylls) and carotenoids (lutein, neoxanthin, *trans*-violaxanthin, *cis*-violaxanthin, antheraxanthin, zeaxanthin and α-cryptoxanthin) in the leaves of *VvmybA1* transgenic 'Hamlin' compared with control wild type.

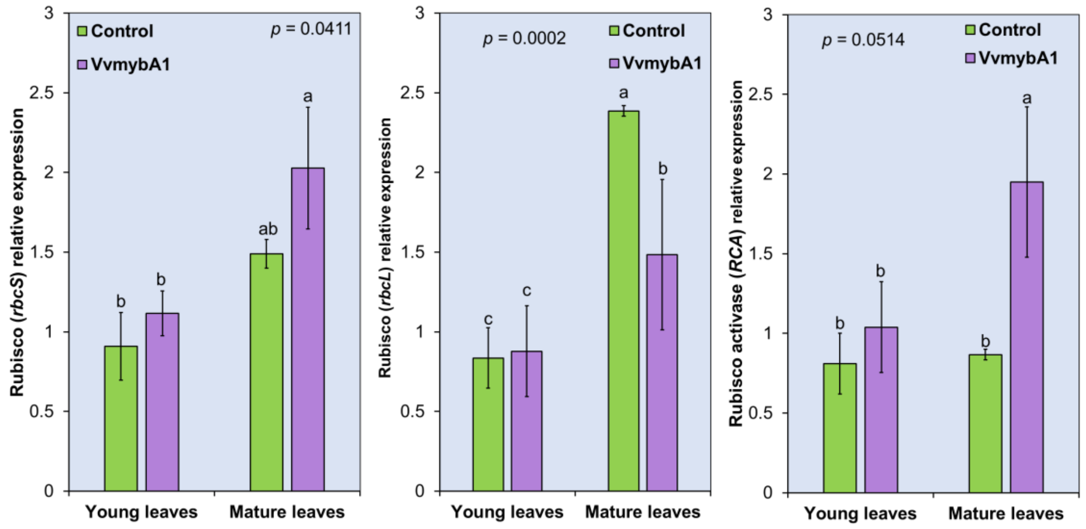

Figure S7. Relative expression of Rubisco transcripts, rbcL: Ribulose-1,5-bisphosphate carboxylase/oxygenase large subunit, *rbcS*: small subunit of Rubisco and RCA: Rubisco activase in the leaves of *VvmybA1* transgenic 'Hamlin' compared with control wild type.
